# What networks in the brain system sustain imagination?

**DOI:** 10.3389/fnetp.2023.1294866

**Published:** 2023-11-01

**Authors:** Riccardo Fesce, Roberto Gatti

**Affiliations:** ^1^ Department of Biomedical Sciences, Humanitas University, Milan, Italy; ^2^ Istituto di Ricovero e Cura a Carattere Scientifico, Humanitas Research Hospital, Milan, Italy

**Keywords:** kinaesthetic motor imagery, visual motor imagery, action observation therapy, imagination, brain networks, consciousness

## Abstract

The brain cannot stop elaborating information. While the circuitries implied in processing sensory information, and those involved in programming and producing movements, have been extensively studied and characterized, what circuits elicit and sustain the endogenous activity (which might be referred to as imaginative activity) has not been clarified to a similar extent. The two areas which have been investigated most intensely are visual and motor imagery. Visual imagery mostly involves the same areas as visual processing and has been studied by having the subject face specific visual imagery tasks that are related to the use of the visual sketchpad as a component of the working memory system. Much less is known about spontaneous, free visual imagination, what circuits drive it, how and why. Motor imagery has been studied with several approaches: the neural circuits activated in the brain during performance of a movement have been compared with those involved in visually or kinaesthetically imagining performing the same movement, or in observing another person performing it. Some networks are similarly activated in these situations, although primary motor neurons are only activated during motor execution. Imagining the execution of an action seems unable to activate circuits involved in eliciting accompanying motor adjustments (such as postural adaptations) that are unconsciously (implicitly) associated to the execution of the movement. A more faithful neuronal activation is obtained through kinaesthetic motor imagination—imagining how it feels to perform the movement. Activation of sensory-motor and mirror systems, elicited by observing another person performing a transitive action, can also recruit circuits that sustain implicit motor responses that normally accompany the overt movement. This last aspect has originated the expanding and promising field of action observation therapy (AOT). The fact that the various kinds of motor imagery differentially involve the various brain networks may offer some hints on what neural networks sustain imagery in general, another activity that has an attentive component—recalling a memory, covertly rehearsing a speech, internally replaying a behaviour—and a vague, implicit component that arises from the freely flowing surfacing of internal images, not driven by intentional, conscious control.

## 1 Introduction

The brain cannot stop elaborating information, whether it originates from sensory paths or from endogenous activity within the brain itself. The circuitries implied in elaborating sensory information have been intensively studied, and most aspects of sensory elaboration by the brain—areas and circuits involved, processing algorithms—are essentially known. The structures involved in programming and producing movements have also been extensively studied and characterized. Much less is known about what circuits elicit and sustain the continuous, spontaneous, endogenous activity of the brain—what might be referred to as imaginative activity. On the one side, whereas perceptual, cognitive, motor, or executive tasks can be standardised, and their performance studied in a controlled, reproducible way, imaginative activity is not equally amenable to systematic study. Nevertheless, two areas of imaginative activity that have been intensely investigated: visual and motor imagery.

Visual imagery mostly involves the same areas as visual processing and has been studied by having the subject undertake specific visual imagery tasks (e.g., mental rotation); these tasks are related to the use of visual imagery as a visuospatial sketchpad, a component of the working memory system. Much less is known about spontaneous, free visual imagination, what circuits drive it, how and why, although networks possibly sustaining specific visuospatial operations (such as self-visualization or scene representation) can be activated and identified in the resting brain (see below).

Motor imagery, on the other hand, may yield more informative hints about the general organization of imaginative activity. Motor behaviour is controlled by distinct brain structures that are differentially involved in intentional movements, inattentive or automatic motor behaviour, or unconscious movements, such as anticipatory or compensatory postural adaptations. Presumably, cognitive behaviour, thought, and imaginative activity are controlled in an analogous way, as their modes and levels of awareness range from attentive reasoning to mind wandering and implicit subconscious processing.

In this paper we review literature about brain networks and motor imagery with the aim of investigating which networks are differentially involved in explicit (conscious) and implicit (essentially unconscious) aspects of mental activity. The approach is based on the following premises:

1. In a very simplified—but quite tenable—way, one could claim that the brain continuously produces electrical activity and elaboration of information. This baseline work, that sustains imaginative activity, is guided only in part by sensory experience or cognitive elaboration by the working memory system; part of it proceeds on its own, with no apparent connection with the momentary situation. We are aware of only a fraction of this activity; such fraction can be reported, and may therefore be defined as “explicit,” while a large fraction remains “implicit,” unconscious (or subconscious, depending on whether a possibility exists to make it explicit and therefore conscious)
2. Several networks have been identified in the brain, based on correlated activity in distinct structures while performing specific tasks. Some of these networks are active at rest, when the subject is not performing any practical or cognitive task; they are often referred to as default mode networks (DMN), and are likely candidates for sustaining imaginative activity, which never stops, and appears to take control of the brain in such resting conditions. Several Authors have tried to dissect sub-networks in this DMN, to identify the circuits specifically implied in the various aspects of basal, resting, brain activity. Still, it is obviously hard to design protocols to experimentally investigate what circuits sustain implicit (unconscious) rather than explicit (conscious) mental activities.
3. Motor imagery has been intensively studied in the last decades, not only because of mere scientific interest in the process, but also because it has gained an increasing relevance in the development of strategies for motor rehabilitation. Motor imagery can be performed in several different ways: imagining performing (1st person, or kinaesthetic) or see somebody else perform (3rd person, or visual) a movement, or observing another person performing it (action observation, AO), or even observing oneself performing it—therefore in a first-person perspective—using virtual reality devices. The brain circuits and networks activated in these different forms of motor imagery have been the subject of many studies, and the differences are instructive; the cortical and motor responses—electromyographic activation of muscles, responses to transcranial magnetic stimulation, interference with the myotatic or H reflexes—have also been examined in these distinct conditions. A major result of the latter studies regards postural adaptation and other “unintentional” movements (that the subject is unaware of) that only occur (or are facilitated) in some of these modalities of motor imagery. This appears to bear some relevance with respect to the therapeutic efficacy; in our perspective, here, this permits us to dissect conditions that do or do not involve an implicit (unintentional and possibly unconscious) component of motor control.

Based on these premises, the comparative investigation of the neural networks activated by the various types of motor imagery, cross-referenced with the analysis of the explicit and implicit components of motor control in these same conditions, may help us to spot and identify neural networks that are differentially involved in explicit or implicit (conscious or unconscious) aspects of imaginative (and mental) activity. In the following sections, we shall recapitulate current knowledge about the distinct brain networks, with a focus on their activation in various forms of motor imagery, to learn what they can teach us about the various levels of awareness that accompany imaginative activity in general.

## 2 Brain networks

During the last 30 years, several brain networks have been identified as sets of cerebral areas that are activated in a coordinated way during specific mental or executive tasks. Coherent fluctuations in activity were observed in specific neuro-anatomical systems, such as the somatomotor system (Biswal et al., 1995) and other distributed networks related to visual or auditory processing (Biswal et al., 1997), to language circuits (Hampson et al., 2002), to executive control, dorsal attention, and salience detection (see. e.g., Smith et al., 2009; Fox and Raichle, 2007). Curiously enough, this whole field of task-related brain networks has received essential contributions from the analysis of the activity of the brain when it is not (at least apparently) performing any task. Any basal, resting activity of the brain had always been considered kind of a disturbing baseline noise, in EEG recording as well as in PET or fMRI imaging: a signal that had to be subtracted and cancelled from the recordings in order to isolate and study the specific activities connected to a task, such as evoked potentials or magnetic responses. In a seminal work, Shulman et al. (1997) noted that a constellation of areas in the human cerebral cortex consistently reduced their activity when performing various goal-directed tasks, compared to a resting state of eyes closed or visual fixation. More precisely, if the oxygen extraction fraction is measured, these areas do not prove to be more active than the rest of the brain, at rest (Raichle et al., 2001); so, they can be considered as areas that are involved, with many others, in a “default” mode of brain function, and are turned down in performing goal-directed tasks. These areas include the precuneus and posterior cingulate cortex (PCn and PCC), in addition to the medial prefrontal cortex (MPF). Greicius et al. (2003) observed intriguing coherence patterns in the fluctuations of activity (<0.1 Hz) among these areas, which were therefore suggested to constitute a connected network, that has been since referred to as default mode network (DMN; for a review, see Raichle, 2015). In a controversial paper, Fox et al. (2005) observed a dichotomy in response to attention-demanding cognitive tasks, involving increased activity in regions whose function supports task execution and decreased activity in regions presumably supporting unrelated or irrelevant processes. These anticorrelations in the resting state between the default mode network on one side, and the so-called dorsal attention network (DAN) and elements of frontoparietal control networks on the other side, suggested that the “default” operating mode of the brain, at rest, involves alternate activation of these two systems, giving rise to periods of mental activity oriented toward the interior and periods of temporo-spatial orientation through the exam of the surrounding environment. The slow alternance of these two systems can be subjectively experimented if one sits in the subway and lets the mind wander: they will realize that even though they are lost in their thoughts, every once in a while, they raise their head and give an explorative look all around. This led Fox et al. (2005) to rename the DMN as “task-negative network.” However, Popa et al. (2009) subsequently showed that during continuous recordings in the cat, although anticorrelations were present for about 20% of the time, correlations spanned the remaining 80% of the time. Therefore, activity in the DMN and the DAN are not mutually exclusive; rather, during increased attentional demands, the DMN would be enhanced in parallel with the DAN.

The default mode network is considered to sustain emotional processing (ventral medial prefrontal cortex, ventral MPF), self-referential mental activity (dorsal MPF), and the recollection of prior experiences (posterior elements of the default mode network). Many authors have associated the default mode network with the mental state of relaxed rest and therefore with the functionality of daydreaming, mind wandering, or stimulus-independent thoughts. However, the brain consumes about 20% of the energy used by the body, and local task-evoked changes in energy use do not exceed 5%. Therefore, either unconstrained thoughts are more energy demanding than are task-oriented ones, or all cognitive functions, whether unconstrained or task-oriented, merely interfere with, and superimpose on, a massive background, spontaneous, implicit, and uninterrupted information processing activity by the brain.

The fact that neural networks intensely operate at rest is not particularly surprising, because any group of neurons, even when cultured in a Petri dish, will rapidly establish a continuous electrical and synaptic activity (Lee and Sheng, 2000). So, the brain would not be expected to ever stop producing synaptic signals and neuronal elaboration, and this would sustain a function that we may not want to call a task, because it is not finalized, but needs anyway to be considered as an activity; an activity that can be probably best defined as imaginative activity.

**FIGURE 1:**
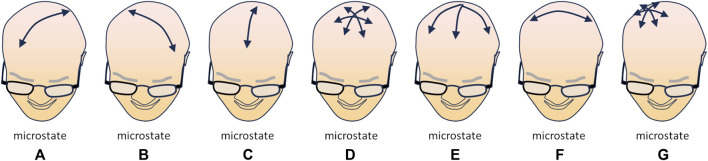
Schematic illustration of the currently identified microstates [**(A–G)**; see text for explanation], i.e., moments in which a specific topology (direction of the dominant polarization) can be observed in multipolar EEG recordings. The arrow(s) in each drawing depict(s) the direction(s) of the main polarization of the EEG signal in the corresponding microstate (redrawn from Figure 3 data in Tarailis et al., 2023).

The study of the resting brain through multipolar EEG has revealed that a set of specific momentary patterns of activity (“microstates”) can be identified and quantified as sub-second time epochs with stable field topography of the power of the signals; this suggests that specific correlations in the intensity of neural activity among various areas of the cortex may represent distinct aspects of mental activity of the resting brain (Koenig et al., 2002). Currently, four—possibly seven—microstates have been characterized: microstate A, characterized by a left-posterior to right-anterior axis, associated with auditory and visual processing and linked to arousal; microstate B, with a right-posterior to left-anterior axis, mostly associated with visual processing related to self, autobiographical memory, scene-visualization; microstate C, with a postero-anterior axis, related to processing self-relevant, self-referential, interior and autonomic information, related according to several Authors to the DMN; microstate D, radially centred on the medial fronto-parietal areas, associated with executive functions, working memory and attention, related to the DAN. In addition to these “standard” microstates, three other have been identified: microstate E, initially merged with microstate C, but radially centred on the postero-medial regions (precuneus and posterior cingulate cortex) and related to processing interoceptive and emotional information and to the salience network; microstate F, centred on the left temporo-parietal areas, possibly involved in processing self-relevant information, mental simulations, and theory of mind; microstate G, centred on the right parietal area, possibly linked to the somatosensory network (Figure 1) (for a systematic review, see Tarailis et al., 2023).

So, the idea that the DMN sustains free imaginative activity, as opposed to the DAN that drives goal-oriented tasks, certainly appears as an oversimplification. Several distinct modes of information processing alternate in the resting brain, and when a task is initiated, the DAN increases its activity together with the task-specific networks, while the DMN is usually turned down, unless increased emotional relevance or attentional demands are involved.

## 3 Motor control and motor imagery

### 3.1 Motor control deploys on at least three distinct levels

#### 3.1.1 A motor plan can be conceived, elaborated, and executed intentionally and attentively

**Data**: Although this is driven by the cerebral cortex, the premotor and motor cortex mostly control the direction and the extension of the movement, rather than the detail of the single muscles that are activated (Georgopoulos, 1994; Schwartz and Moran, 2000). So, while one intentionally performs a movement—e.g., reaching forward with a hand—and can explicitly tell what they are doing, non-conscious elaborations are at work: the cerebellum, with the help of error detecting circuits in the inferior olivary complex, gives back a detailed feedback to the cerebral cortex (Li and Mrsic-Flogel, 2020), and tunes the subcortical descending systems, so that all the muscles involved in the movement are activated with absolute precise timing and intensity (Benagiano et al., 2018). Simply pushing forward the hand involves muscles in the shoulder, the arm and the forearm, and the correct acceleration and deceleration of the hand require proper timing and intensity in the activation of agonists and antagonists, to stop the movement at the right time, so that the hand does not overreach. Thus, although the intentional movement is explicit, the exact detail of its execution is not.

**Speculation**: The involvement of these subcortical circuits for implicit detailed programming is certainly needed in executing a motor act, but possibly not called for in visually imagining oneself performing the same act (you just imagine your hand moving forward). As regards the cortex, one may expect some activation of premotor areas, whether the movement is executed, imagined, or observed, but activity in the primary motor cortex only when the movement is executed.

#### 3.1.2 Motor activity—most of what we do—can occur in a conscious but inattentive, and possibly unintentional, mode

**Data**: All the elements of the surrounding environment—conditions and objects—are perceived through sensory systems and are elaborated in the so-called ventral stream (temporal lobe) with the aim of interpreting shapes, sounds and other stimuli to identify objects (the so-called “what pathway”; Tootell et al., 1997), while in the so-called dorsal stream (parietal lobe) the spatial relations are analysed, among objects and between oneself and the object. The two streams of information converge in the hippocampus, which also receives intense input from subcortical and limbic areas involved in elaborating the vital and emotional relevance of the sensory information. The hippocampus contextualizes objects and events into an integrated and affectively coloured perception of the situation (a subjective experience) that is relayed to higher associative cortices (Eichenbaum, 2017). The dorsal stream, however, does not have a sensory function only: stimuli and objects, localized in space with respect to oneself, are perceived according to the way they present themselves and can be interacted with. The “affordance” of the object is appreciated in the parietal cortex, which makes this area a sensory-motor hub rather than a mere sensory area (Maranesi et al., 2014). The possible interactions with all the elements and objects of the scene are prompted to the premotor areas as possible behaviours. They are not enacted, however, because the feedback loop of the basal nuclei tends to inhibit every action proposed by the premotor cortex. Every possible interaction with an object may have been performed in the past (and therefore be associated with a variable vital, affective, hedonic, and operational valence) or may suggest possible positive or negative outcomes (Averbeck and Costa, 2017); these aspects are translated into variable dopamine release by the substantia nigra onto each specific circuit of the dorsal striatum, so that only the actions that look “promising,” or have been successful and therefore positively reinforced in the past, will be performed. This form of behaviour—which we may refer to as “autopilot” behaviour—accounts for most of the motor behaviour of animals, but also for a large fraction of human behaviour: these motor activities certainly are conscious but are not attentively and intentionally monitored while they are performed. Obviously, these activities also exploit the fine control by the cerebellum, so that complex tasks can be performed in a fully automatic way: since the cerebellar circuits are quite rapid and do not need cognitive monitoring, activities performed in such automatic way can be executed with much higher speed and accuracy than under attentive control. So, behaviours performed under “autopilot” and “automatic” control have even larger implicit components (Jueptner and Weiller, 1998).

**Speculation**: During motor execution, activation of the somatosensory cortex by proprioceptive feedback, and some involvement of subcortical structures, are expected in motor execution; they may also occur in kinaesthetic motor imaging, as the subject is invited to “feel” the movement; in pure visual motor imagery they are not expected, but some activation might occur during action observation, thanks to the involvement of the mirror neuron system, that is supposed to trigger an “embodied simulation” of the observed action. As regards the basal nuclei, the control and supervision by the inhibitory circuitry is presumably needed when intentionally imagining a movement, to avoid actually executing it; on the other hand, when the action is observed (AOT), rather than imagined, this action is not supposed to be needed.

#### 3.1.3 Postural adaptations accompany every motor act that tends to produce some shift in the body centre of mass

**Data**: Although we typically meet Newton third law—forces always act in equal but opposite pairs, and for every action, there is an equal but opposite reaction—in high school, it seems that our motor control systems are well-aware of this principle on their own. In fact, almost every motor act tends to produce some shift in the body centre of mass. Imagine a chest pass, the act through which a basketball player passes the ball to a teammate: since one is pushing the ball in front of them, the body necessarily experiences a pushback. Still, a quite sophisticated system of preparatory and compensatory postural adaptations avoids any impairment of balance. This similarly occurs for every action, even when the forces involved are much weaker. One is absolutely unaware of these adaptations, but their occurrence can obviously be monitored and recorded electromyographically or through neuroimaging techniques. As discussed below, these unconscious responses can be activated not only when a movement is executed, but also, under some conditions, when the movement is only imagined.

**Speculation**: very few studies investigated the neural mechanisms of anticipatory postural adaptations (APA) (Ng et al., 2013) and what circuits specifically control these responses remains an open issue. Several studies have identified the circuitries differentially involved in attentive and purposeful actions, in spontaneous or inattentive behaviours, and in implicit, subconscious motor adaptations, using EMG, EEG, PET, fMRI and TMS. Given the growing interest for motor imagery and action observation therapy for motor training and rehabilitation, research is expanding on similar investigation of the circuits involved in corresponding modes of motor imagery. The growing evidence in this field offers unprecedented hints in understanding how imaginative activity works in general, and what circuits may sustain its various modes and forms.

### 3.2 Motor imagery

Several aspects of motor imagination have been studied in detail. The neural circuits activated in the brain during actual performance of a movement have been compared with those involved in imagining performing the same movement, or in observing another person performing it. Some networks are activated in quite the same way in the three situations, although the circuits involved in executing the movement (e.g., primary motor neurons) remain silent. However, imagining performing an action seems unable to activate circuits involved in eliciting accompanying motor adjustments (such as postural adaptations) that are unconsciously associated to the performance of a movement, as if they were an implicit component of the motor scheme. A more faithful neuronal activation seems to be obtained through the so-called kinaesthetic motor imagination—the subject being invited to imagine all the proprioceptive components of (how it feels to perform) the actual movement. Activation of premotor and mirror circuits, which sustain not only the overt movement but also the implicit accompanying adaptations, can be elicited by observing another person performing an action. This last aspect presumably accounts for a long-known phenomenon in sports training, that the mere observation of an athletic gesture correctly performed was “magically” able to improve the performance of athletes. The idea of using this approach in motor rehabilitation and physiotherapy was conceived by Ertelt et al. (2007), and has originated the expanding field of AOT—action observation therapy. AOT exploits the frontoparietal network of mirror neurons system, mainly located in the ventral premotor cortex, inferior parietal lobule, and inferior frontal gyrus. During AOT, patients are asked to observe videos of goal-oriented movements, realized based on their motor impairment, and asked to reproduce the movements. This approach has been investigated for its potential benefits in several neurologic and musculoskeletal diseases (Ryan et al., 2021).

It appears that the distinct kinds of motor imagery—programming (explicitly) an action, or actively (explicitly), or holistically (implicitly) imagining the same action—differentially involve the various brain networks (Dietrich, 2008). This may constitute a revealing paradigm in investigating the neural networks that sustain imagery in general. In fact, the latter has an active, oriented component—recalling a memory, covertly rehearsing a speech, internally replaying a behaviour—and a vague, implicit component that arises from the freely flowing surfacing of internal images (sensory, coenesthetic, emotional, conceptual), not driven by intentional, conscious control. Identifying the network substrates of these differential activities would help clarifying how the freely flowing, nonverbal, vague, and ambiguous wandering of imagination can be intentionally and actively crystallized into an explicit and precise line of thought.

Motor imagery, in particular active motor imagination and action observation, constitute a particularly interesting field, because the involvement of the various brain structures in the distinct motor-related mental activities can be examined, and the activation of distinct network can be correlated with the degree of intentionality, awareness and attentive cognitive control, with the degree of precision of the movement, and with the possible engagement of other normally associated and coordinated movements and postural adaptations.

In a provocative article, Dietrich (2008) questions the validity of those studies that, given the impossibility of applying extensive neuroimaging approaches to performing athletes, try to understand the central control of athlete motor performances by using PET, CAT scans, EEG, and fMRI on them while they are imagining performing. The intriguing aspect is that the Author does not make his point about the fact that there is no actual movement, and therefore there will be various brain areas that are or are not activated; rather, his argument consist in the fact that the intentional, explicit imagination of the movement does not activate the same complete pattern of accessory movements and postural adaptations that the actual spontaneous movement elicits, especially if it is a well known, trained, motor behaviour.

This discrepancy is well documented in a meta-analysis on cortical activations detected by fMRI or PET during motor imagery, action observation, and movement execution (Ref. Hardwick et al., 2018). The networks activated in the three conditions were partly specific and partly shared. Motor imagery recruited a network of bilateral cortical and subcortical regions: premotor, inferior and superior parietal, left dorsolateral prefrontal, SMA and cingulate cortices, putamen, and cerebellum. Action observation recruited a bilateral network which included dorsal and ventral premotor cortices, pre-SMA, right superior occipital gyrus, superior and inferior parietal lobule, and the occipital cortex (but not the primary, Broadman area 17, or the lateral geniculate nucleus, directly involved in actual vision). Interestingly, this condition did not activate subcortical regions. During movement execution sensorimotor and premotor cortices were activated, while subcortical activations were identified in the bilateral thalamus, putamen, and cerebellum.

A contrast analysis aimed to compare motor imagery with action observation showed that motor imagery was associated with recruiting premotor regions, including bilateral SMA, PMd, and PMv, bilateral areas of the inferior parietal lobe, and regions of the left superior parietal lobe and DLPFC. As expected, the cortical areas in which mirror neurons have been identified (bilateral inferior frontal gyrus and right inferior/superior parietal lobule) were more associated with action observation.

It is necessary here to clarify that motor imagery is a tricky business, because there are at least 3–4 substantially different ways in which a movement can be “imagined”: motor imagery can be elicited by asking the subject to imagine performing an action (intentional, explicit imagination), or by asking them to imagine the experience of (what they feel in) performing the action (a kinaesthetic imagination), or by exposing the subject to a movie (or a virtual reality representation) showing the action of interest, either from the third-person (visual motor imagery) or in the first-person perspective (closer to kinaesthetic imagery).

Interestingly, the neural networks that are activated by these procedures only partially coincide, suggesting that two distinct networks can be identified: one more explicit, linked to conscious awareness and accessible to verbal reporting, and an implicit system, based on skill or experience, only partially accessible to conscious reporting (Dietrich, 2008).

Electrophysiological data, acquired using a 64-channel EEG, showed that networks activated during kinaesthetic motor imagery resulted more similar to those active during motor execution than during visual motor imagery. The correlation coefficients showed similar connectivity patterns between kinaesthetic motor imagery and motor execution, as well as between visual motor imagery and action observation. Moreover, the degree centrality of nodes (number of connections onto them) in the somatosensory area (S1) was higher during kinaesthetic motor imagery than during visual motor imagery; conversely, a higher number of connections were observed during visual motor imagery in the right premotor cortex, involved in planning and preparation of actual movements (Yang et al., 2021). Interestingly, only kinaesthetic motor imagery seems to be able to increase corticospinal excitability (Stinear et al., 2006) through of activation of M1, as detected through MEG (Schnitzler et al., 1997), EEG (Caldara et al., 2004), fMRI (Porro et al., 2000; Kuhtz-Buschbeck et al., 2003), and TMS (Fadiga et al., 1999).

Studies on brain networks involved in motor imagery led to a physiotherapy approach named graded motor imagery (GMI) addressed to decrease chronic pain (Moseley, 2006). Its rationale consists in inducing a gradual activation of brain networks related to painful body parts. GMI consists in three distinct stages. In the first stage, patients must discriminate between left and right body parts showed to them. This cognitive exercise promotes activation of bilateral superior parietal lobules and visual extrastriate cortex (Vingerhoets et al., 2002). The second stage involves explicit motor imagery to train the brain to represent the movement causing pain, without inducing it by actual movement. In the last step, the illusion is created that the painful body area is moving without inducing pain by placing a mirror so that it masks the painful part and reflects the movement of the contralateral, unaffected body parts. Interestingly, this last stage of GMI seems to be able to involve the activation of the primary motor cortex, M1 (Lee et al., 2015)

### 3.3 Explicit and implicit systems

The existence of an explicit and an implicit system was thoroughly discussed with reference to memory; the two systems can be dissociated from each other functionally and anatomically (Squire, 1992; Schacter and Buckner, 1998). As regards psychomotor learning, it is considered as an implicit form of memory, although one may be able to explicitly recall and describe the movements involved.

In the clinical practice of motor rehabilitation, the relative importance of these two systems has acquired a paramount importance. Significant differences are observed in terms of motor recovery depending on whether the patient is invited to perform a specific movement or a finalized action, or to imagine performing the same action, to imagine what they feel in performing the action, to observe the same action performed by someone else or to observing themselves apparently performing the action, through a virtual reality device (or using an appropriately positioned mirror while performing the action with the contralateral limb).

The whole field of AOT, action observation therapy, has stemmed from these observations, given the unexpectedly promising efficiency of the procedure (and the large interest in the mirror neurons and the functions of the mirror mechanisms in the brain).

There are two main differences between explicit and implicit systems in brain function: at difference with implicit elaboration, explicit conscious awareness is a higher order kind of information (meta-information), as it also contains information about its own content; on the other hand, the structure of the nervous system is such that every bit of information is simultaneously fed to many distinct circuits that elaborate it, by putting it in relation with different sets of data; the result of such elaboration can be accessed and reported explicitly, but the whole elaborative process, in all its richness and complexity, cannot, and will remain implicit; thus, the scope of explicit consciousness is necessarily much restricted with respect to the scope of implicit knowledge.

It is generally thought that a mental content accessing conscious awareness consists in the corresponding neural activity being able to interact with endogenous (spontaneous) activity and recruit other circuits; it would thus expand in time and space to generate a resonance in a diffuse brain network (temporo-spatial theory of consciousness; Northoff and Zilio, 2022), and end up being broadcasted in the global neuronal workspace (GNW; Baars and Franklin, 2009). Access to consciousness is therefore essentially a matter of gaining selective attention. The process of selective attention is based on the competition between two components: a bottom-up selection mechanisms, based on the vital, hedonic, emotional, affective relevance of the mental content; and top-down mechanisms, based on its connection and bearing on what is currently being elaborated by the working memory system (Knudsen, 2018). On the other hand, in addition to the selection of the content, explicit awareness requires extracting a clear, unambiguous, reportable synthesis of such content, i.e., the result of a discriminative elaboration of the corresponding neuronal activity. Discriminative elaboration by the brain requires that the fundamental elaborative units of the cerebral cortex—the pyramidal neurons—precisely recognize the patterns of simultaneous synaptic activations they receive; this is not possible if inputs that occur more than 1 ms apart are able to sum onto each other. The window for temporal summation of inputs on the pyramidal neurons can be maintained at about 1 ms if each input that reaches the cell is also received by an inhibitory interneuron, which will rapidly shut off the response of the pyramidal cell through its inhibitory action. A specific circuit in the cortex can operate in this discriminative way only if it is kept active—by projections from the brainstem and the basal forebrain—and maintains the corresponding projecting neurons in the thalamus slightly depolarized; this way, information can be precisely relayed by the thalamus to the cortex. If instead the cortical circuit does not maintain the corresponding thalamic neurons slightly depolarized, these will tend to emit bursts of spikes, which will fatigue and eventually switch off the inhibitory interneurons, so that the activations of the pyramidal cell become more prolonged, the time window for summing synaptic inputs is prolonged, and the elaboration becomes smeared, approximated, non-discriminative (Tremblay et al., 2016).

An important correlate of what has just been said is that the more discriminatively an area of the cortex is operating, the less the activity of neighbouring neurons will tend to be synchronized, and the frequency composition of the local EEG will tend to shift to higher frequencies. This is typically observed on occipital-parietal areas, which are dominated by alpha rhythms with eyes closed, and are desynchronized, shifting to higher frequency beta rhythms, when the eyes open and visual information starts being elaborated. Similarly alpha rhythms in premotor and motor areas (generally referred to as mu waves) tend to desynchronize and wane when motor activities are programmed or executed [event-related desynchronization (ERD); see, e.g., Inamoto et al., 2023].

**FIGURE 2:**
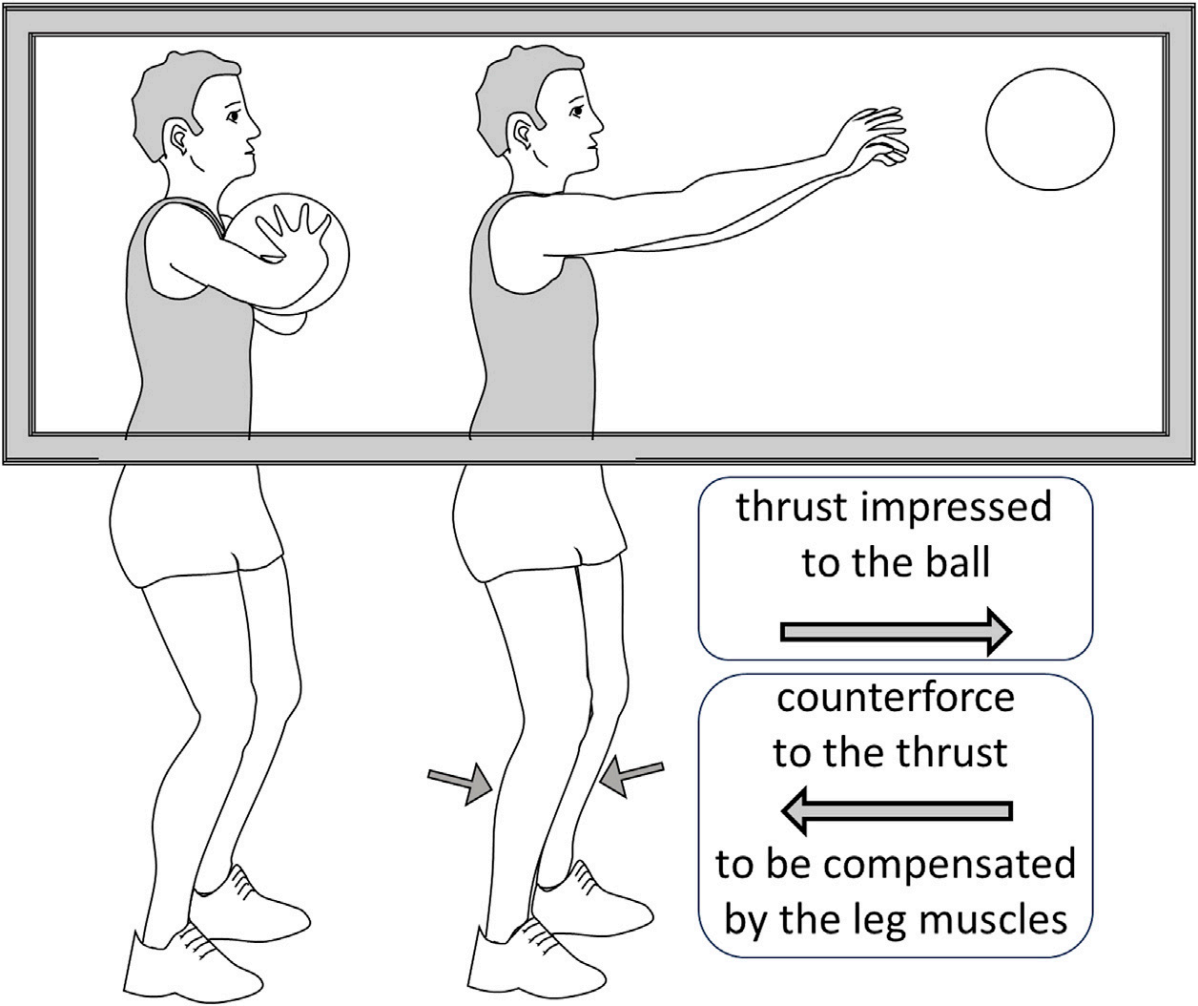
A depiction of the observation by Bolzoni et al. (2023). The subject is shown a basketball player performing a chest-pass, a gesture that requires an anticipatory postural adaptation by activating the leg muscles to compensate the counterforce produced by the thrust impressed to the ball. Although the subject can only see the upper part of the body, the reflexes in the calf muscles are potentiated, indicating that the descending system has predisposed them for the anticipatory adaptation.

If we focus on motor imagery and motor performance, all this suggests that being discriminative, the explicit, intentional imagination of a motor behaviour—such as throwing a basketball, or performing a purposeless movement—should not be able to evoke the whole complexity of the act (including implicit postural adaptations), as opposed to engaging the implicit knowledge of the movement through mirror neurons—when seeing another subject that throws the baseball—or by imagining an action (a finalized behaviour) rather than the corresponding movement. Conversely, one should expect that a defect in the discriminative elaboration of a behaviour may make it difficult to isolate and produce a precise movement, such as moving a hand forward, without involving a set of other movements that tend to accompany the requested movement. This is what typically occurs following cortical damages in motor areas (Choi et al., 2023); consistently, proposing a transitive action—which will not need to be studied in its kinematics, but simply executed by activating implicit schemes—turns out to be much more effective in training and rehabilitating a stroke patient (Sullivan, 2007).According to Dietrich (2008), visual motor imagery is a conscious process that requires the engagement of the explicit system. Conversely, actual, purposeful motion is controlled by the implicit system, which we know is implemented in a set of anatomically distinct brain structures. Kinaesthetic motor imagery is likely to be controlled by (or at least significantly involve) the implicit system, and the same may be true for action observation, which is able to activate the mirror neuron systems related to unconscious postural adjustments (Figure 2) (Bolzoni et al., 2023). In this scenario, the scarce activation of subcortical structures during action observation might be due to the fact that the fine coordination of distinct muscular groups to precisely control and correct the movement is not needed. A related, interesting issue regards the role of the observer’s acquired motor skills in the relation between implicit and explicit components of actions: Calvo-Merino et al. (2005) had ballet and capoeira dancers to observe movies reporting either type of dance, and subjects displayed stronger brain activations in the areas classically associated to mirror systems when they observed movement belonging to their personal motor repertoire; this suggests that mirror neurons, involved in producing a covert embodied simulation, are more active when the observer is familiar with the implicit components that significantly contribute to the observed (or kinaesthetically imagined) motor behaviour.

The interesting question here—for its relevance both in the field of physical rehabilitation and in underpinning the structural substrates of explicit and implicit consciousness—is to investigate whether the distinction is properly the one just mentioned, between imagination and actual performance of a movement, or the involvement of the various brain networks points to a much more complex picture: a picture in which differential brain network are activated when comparing purposeless movement to finalized actions; kinematic to kinaesthetic imagination of movements; or imagination to observation; and so on.

## 4 Cognitive behaviour vs. motor behaviour

The distinction proposed here between explicit and implicit motor control applies rather precisely to cognitive behaviour as well. Motor behaviour is driven by two main mechanisms: external cues suggest possible interactions, while internally developed strategies ask for oriented behaviours. The selection among possible behaviours is determined by a bottom-up drive, consisting in the vital and hedonic relevance of each behaviour and its possible outcomes, and a top-down control, which privileges behaviours functional to current purposes or (short- or long-term) strategical objectives. A large fraction of motor behaviour proceeds in a “spontaneous,” unattended (unintentional), “autopilot” way. Mental activity is similarly guided by the mechanisms of selective attention (Knudsen, 2018): bottom-up request of attention based on vital/emotional relevance, and top-down control based on the relatedness to the current line of thought. Like motor behaviour, cognitive behaviour largely proceeds on an undirected, possibly semi-conscious or implicit, level.

There are two main kinds of implicit components in both motor and cognitive behaviour.

One is an implicit contribution to intentionally initiated movements, that consists in inadvertently refining, enriching, adjusting, and correcting the movements and producing fluid sequences of appropriate movements; in the cognitive domain, this is paralleled by the unattended transformation of a preverbal concept into well-formed, grammatically correct, and fluidly flowing sentences. These functions are performed by the cerebellum and the basal nuclei. The cerebellum translates the cortical request of a movement into the precise timing and intensity of activation of each muscular groups that participates in the movement, checks and corrects the ongoing movement in real time, and learns to reproduce behaviours in an automatic way, with speed and precision than cannot be attained under attentional control by the cerebral cortex (Therrien and Bastian, 2019). The basal nuclei, an inhibitory circuitry, are modulated by midbrain dopaminergic centres to favour movement initiation, to switch from one movement to another, and to select among possible behaviours. This controls our unattended, “autopilot” behaviour: since the parietal cortex elaborates the affordance of every object that we perceive and triggers the motor schemes for appropriate interaction with the object in the premotor cortex (dorsolateral, cue-drive path of motor control), inhibition and choice by the basal nuclei are indispensable to guide an efficient unattended behaviour (Lanciego et al., 2012). The putamen also regulates the speed of movements.

Although these subcortical structures were traditionally associated almost exclusively to motor control, it is now clear that they largely contribute to cognitive activity as well (Koziol, 2014): the cerebellum takes care of ordering of the words in a sentence, associating the appropriate person and tense of verbs, and modifying words for singular/plural (for gender in some languages); also, it learns to guide the automatic repetition of poems, prayers, and texts learnt by heart. The basal nuclei guide the line of thought and selective attention the same way they guide movement selection and fluid flow; patients with Parkinson’s disease display slowed down motor activities and similar increases in the duration of imagined movements (Helmich et al., 2007); Tourette patients display accelerated motor as well as cognitive behaviour (Avanzino et al., 2016). Also, Parkinson’s patients face significant difficulty in switching from a motor sequence to a different one and encounter the same difficulty in producing a mental frame shift.

The other implicit component of motor behaviour involves autonomous movements that we execute in association or preparation to any sensory or motor activity that may interfere—or suggest a future interference—with body balance, i.e., the compensatory or anticipatory postural adjustments that we are essentially unaware of. These do not interfere with the movement, to modify or correct it, but consist in the independent facilitation or activation of specific muscular groups. Freely flowing imagination constitutes a similarly unattended, autonomous, and implicit (possibly unconscious) activity in the cognitive domain.

## 5 Conclusion

What can the networks involved in motor execution and imagery tell us about the relations between explicit and implicit behaviours?

In synthesis, four types of tasks can be considered: motor execution (ME), kinaesthetic motor imagery (KMI), visual motor imagery (VMI, either in the first or in the third person), and action observation (AO, again in either the first or the third person).

Obviously, relevant activation of the occipital lobes was observed in visual imagery as well as action observation, as both VMI and AO involve visual processing. Also, both tasks activated parietal and premotor areas, the dorsolateral path of cue-guided, unattended motor control in response to visual (imagined or observed) cues. However, whereas VMI mostly activated a network corresponding to microstate “A” (left posterior to right anterior axis; audio-visual and related to arousal), AO activated a network closer to microstate “B” (right posterior to left anterior, related to visual elaboration of the self, scene representation and autoreferential processing); in particular, at difference with VMI, AO particularly activated regions in which mirror-neurons are thought to be located, namely, bilateral areas in the inferior frontal gyrus (premotor) and areas of the right inferior and superior parietal lobule (Hardwi), that correspond to the above-described microstate “G”, related to somatosensory activity; conversely VMI, but not AO, activated subcortical structures, suggesting that the movement captured and reproposed by the cortex, during action observation, did not need to be properly temporized (putamen) or refined, controlled and corrected (cerebellum). VMI also produced a left-lateralized recruitment of the dorsolateral prefrontal cortex (Hardwick et al., 2018), areas generally involved in executive functions and working memory control (task-oriented motor and cognitive activities).

Cortical activation by KMI resembled ME most closely, activating sensorimotor and premotor cortices; in addition, ME also activated portions of the thalamus, putamen, and cerebellum. An intriguing general observation is that the alpha band dominates in the total EEG power in motor imagery (both KMI and VMI), while the beta band dominates in ME and AO (Yang et al., 2021), consistent with the decrease in mu rhythms (frequency band coincident with alpha) that accompanies motor activation (Inamoto et al., 2023). Still, activation in the primary motor cortex is only observed during motor execution, and not during action observation.

A major difference among the tasks here discussed consists in the capability of activating implicit, unintentional and unattended postural adaptations: motor execution is invariably accompanied by the appropriate anticipatory and compensatory postural adaptations. Kinaesthetic motor imagery can partly activate the same responses (Stinear et al., 2006), suggesting that the sensory-motor component, which is particularly activated in this form of motor imagery, is strongly involved triggering these complementary but autonomous (and unconscious) motor behaviours. Visual motor imagery in the first-person perspective is often considered as a form of KMI; VMI strictly speaking, in the third-person perspective, does not appear to be able to trigger any postural adaptation. It is tempting to suggest that reducing the activity in the network that sustains microstate “A” (audio-visual arousal), mostly active in VMI, and activation of the left dorsolateral areas, may be needed to release implicit motor components, and may similarly be needed to release freely flowing, preverbal imagination.

Action observation, which at first sight would appear to be the task most removed from motor execution, is instead able to trigger postural adaptations (Bolzoni et al., 2023). This is intriguing, as the somatosensory areas are not directly activated by sensory inputs during this task. We may suggest that AO, by activating the mirror neuron system, bypasses the need of strong sensory inputs, and is able to activate somatosensory areas by producing an embodied simulation of the observed action, and, as a consequence, the appropriate cohort of accompanying adjustments and anticipatory and compensatory postural adaptations.

**FIGURE 3:**
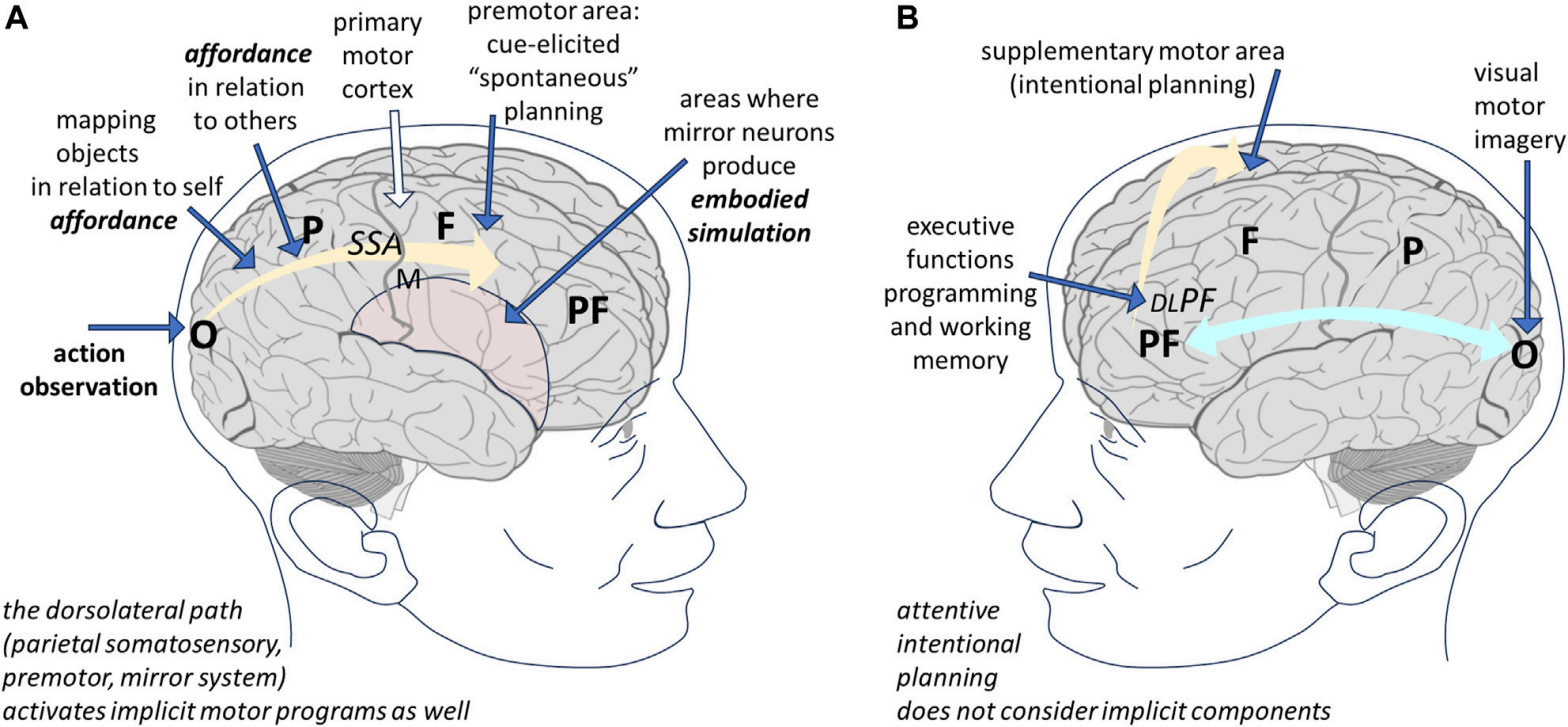
During action observation **(A)**, the parietal cortex maps the affordance of objects for the other person (and the other person’s relation with the environment); as a consequence, the mirror system simulates the somatosensory and proprioceptive experience and the motor programs involved in cue-elicited spontaneous behaviour. This way, the implicit components of the movement—such as anticipatory postural adaptations—are also planned, though they are not executed (white arrow to the primary motor cortex). In visual motor imagery **(B)**, the subject is invited to visualize the action in their mind; this is an executive task, involving conscious elaboration of an intentional behaviour: in this case the programmed motor behaviour does not include implicit components. O, occipital lobe; P, parietal; F, frontal; PF, prefrontal; SSA, somatosensory area; M, primary motor cortex; DLPF, dorsolateral prefrontal cortex (executive functions).

Overall, it seems that either the somatosensory or the mirror neuron systems must be activated in order to trigger the execution of the implicit components of complex motor behaviours. If a parallel can be drawn to the cognitive behaviour, this suggests that the free flow of cognitive activity (unconstrained and unoriented imagination) can predominate when the dorsolateral path, activated by somatosensory (kinaesthetic) and/or external cues and guided in an unattended way by the basal nuclei, prevails on the prefrontal central executive of working memory and the strategical elaboration by the ventromedial frontal cortex (Figure 3).

This is quite reasonable, as in this situation the top-down control of selective attention would be weakened, and release “spontaneous” activities guided by the bottom-up component of attentional control in an unattended, unintentional way (and similarly release implicit components of the movement).

### 5.1 A merely speculative note

In motor rehabilitation, as well as in sports training, winning strategies seem to require that the entire cohort of accompanying and adaptive (explicit and implicit) movements be executed, either actually—when possible—or mentally, through kinaesthetic rather than visual motor imagery or through action observation, of aimed actions rather than mere movements, if possible. An intriguing parallel may be drawn with psychotherapeutic practices: free associations, spontaneous or guided imaginative production, and behavioural conditioning have been widely—and often successfully—used to bypass the attentive cognitive control and try to access the unconscious control of behaviour. Establishing—or restoring—a functional behaviour, be it to improve motor performance or to overcome dysfunctional behaviours or psychological attitudes, seems to require that attentive, cognitive control be weakened, so that the dynamics of implicit (unconscious) brain activities are activated and—sometimes successfully—restored or profitably modified.

## Data Availability

The original contributions presented in the study are included in the article/supplementary material, further inquiries can be directed to the corresponding author.
